# Prevention of Cognitive Decline in Alzheimer’s Disease by Novel Antioxidative Supplements

**DOI:** 10.3390/ijms21061974

**Published:** 2020-03-13

**Authors:** Koh Tadokoro, Yasuyuki Ohta, Haruhiko Inufusa, Alan Foo Nyuk Loon, Koji Abe

**Affiliations:** 1 Department of Neurology, Graduate School of Medicine, Dentistry and Pharmaceutical Sciences, Okayama University, 2-5-1 Shikata-cho, Kita-ku, Okayama 700-8558, Japan; 2 Division of Anti-Oxidant Research, Life Science Research Center, Gifu University, 1-1 Yanagido, Gifu 501-1193, Japan; 3 Hovid Berhad, 121, Jalan Tunku Abdul Rahman, Ipoh 30010, Perak, Malaysia

**Keywords:** Alzheimer’s disease, oxidative stress, supplement

## Abstract

Oxidative stress plays a crucial role in Alzheimer’s disease (AD) from its prodromal stage of mild cognitive impairment. There is an interplay between oxidative stress and the amyloid β (Aβ) cascade via various mechanisms including mitochondrial dysfunction, lipid peroxidation, protein oxidation, glycoxidation, deoxyribonucleotide acid damage, altered antioxidant defense, impaired amyloid clearance, inflammation and chronic cerebral hypoperfusion. Based on findings that indicate that oxidative stress plays a major role in AD, oxidative stress has been considered as a therapeutic target of AD. In spite of favorable preclinical study outcomes, previous antioxidative components, including a single antioxidative supplement such as vitamin C, vitamin E or their mixtures, did not clearly show any therapeutic effect on cognitive decline in AD. However, novel antioxidative supplements can be beneficial for AD patients. In this review, we summarize the interplay between oxidative stress and the Aβ cascade, and introduce novel antioxidative supplements expected to prevent cognitive decline in AD.

## 1. Introduction

The number of dementia patients is rapidly increasing in aging societies. Over 46 million people lived with dementia worldwide in 2015, and this number is estimated to increase to 131.5 million by 2050 [1]. Alzheimer’s disease (AD) is the most common cause of dementia. Of all dementia patients in our clinic, 62% had AD, and 69% of patients were in the late elderly (≥75 years old) subgroup [2]. There is a great demand for effective interventions to prevent cognitive decline in AD, but no effective drugs or supplemental therapies have yet been established.

AD is pathologically characterized by the presence of hallmark lesions such as neuronal loss, and senile plaque consisting of amyloid β (Aβ) and neurofibrillary tangles (NFT). In the Aβ hypothesis, Aβ is widely regarded as a primary cause of cognitive decline. Aβ peptides are cleaved from amyloid precursor protein (APP), a transmembrane protein associated with neuronal development, neurite outgrowth and axonal transport, and released outside the cell, where they are rapidly degraded or removed. Aβ monomers aggregate into oligomers, protofibrils and amyloid fibrils. Although Aβ is rapidly degraded or removed in normal subjects, under pathological conditions, Aβ peptides can accumulate to produce Aβ oligomers, protofibrils or fibrils [3]. Soluble oligomers or protofibrils are supposed to cause neuronal dysfunction including synaptic impairment/spine changes, dendritic simplification, axonopathy/neuronal loss and subsequent memory impairment in AD rather than end-stage amyloid fibrils [4,5]. In patients carrying a mutation for autosomal dominant AD (described below), changes of pathophysiological conditions preceded their cognitive decline; Aβ_42_ in the cerebrospinal fluid (CSF) appeared to decline 25 years before the onset of symptoms, followed by fibrillar Aβ in positron emission tomography (PET), increased tau in the CSF, hippocampal atrophy and hypometabolism [6].

In familial cases of early-onset AD, autosomal dominant mutations of *APP*, *PSEN1* and *PSEN2* have been identified, and the global prevalence for autosomal dominant forms of early-onset AD is 5–10% [7]. These mutations lead to the accumulation of Aβ and subsequent development of AD. On the other hand, in sporadic cases of AD, the mechanism of Aβ accumulation in the brain remains unclear. There are several genetic and non-genetic risk factors of late-onset AD. The apolipoprotein E (ApoE) ε4 allele is a well-known genetic risk factor of AD [8]. In addition, genome-wide association studies identified susceptibility loci such as *CLU*, *PICALM*, *CR1* and *BIN1*, mainly clustered according to their immune response, APP processing and lipid metabolism and endocytosis [9]. Among non-genetic factors, cerebrovascular disease, hypertension, diabetes mellitus, both low and high body weight, dyslipidemia, metabolic syndrome, smoking and traumatic brain injury increase the risk of AD [10].

## 2. Oxidative Stress and Alzheimer’s Disease

### 2.1. Oxidative Stress

Oxidative stress is a disturbance in the balance between the production of reactive oxygen species (ROS) or reactive nitrogen species (RNS) and antioxidant systems in the body [11]. ROS is a type of unstable molecule that contains oxygen and easily reacts with other molecules, including the superoxide anion (O_2_^•−^), hydrogen peroxide (H_2_O_2_) and the hydroxyl radical (^•^OH). The sources of ROS are mitochondria, oxidases (such as nicotinamide adenine dinucleotide phosphate (NADPH) oxidase and xanthine oxidase), and autoxidation of different small molecules of endogenous and exogenous origin [12]. RNS are a family of molecules derived from nitric oxide (NO^•^) and O_2_^•−^ produced via NO^•^ synthase and several enzymes including NADPH oxidase, xanthine oxidase, lipoxygenase and cyclooxygenase [13]. Antioxidants serve to counterbalance the effect of oxidants, and can be classified into enzymatic and non-enzymatic groups. Enzymatic antioxidants include superoxide dismutase (SOD), catalase, glutathione peroxidase (GPx), thioredoxin, thioredoxin, peroxiredoxin and glutathione-*S*-transferase (GST), commonly requiring NADPH as a reducing equivalent. Non-enzymatic antioxidants include low-molecular weight compounds such as vitamins C and E, β-carotene, uric acid and glutathione (GSH). Several studies proved that ROS modulate intracellular transduction pathways and transcriptional factors involved in cell proliferation, differentiation and maturation [14]. However, when ROS accumulation exceeds antioxidant defense, it is referred to as oxidative stress, and is related to pathological conditions [15].

The nervous system is vulnerable to oxidative stress because of its high consumption of oxygen, a large amount of polyunsaturated fatty acids and high iron content resulting in an increased generation of ROS. Therefore, oxidative stress plays a crucial role in various diseases of the nervous system including ischemic stroke, as well as neurodegenerative diseases including amyotrophic lateral sclerosis (ALS), Parkinson’s disease, the prodromal stage of mild cognitive impairment (MCI) and AD [16]. Many previous studies revealed increased free radical production, lipid peroxidation, oxidative protein damage, decreased adenosine triphosphate (ATP) production and reduced cell viability in postmortem AD brains. Praticò et al. noted that individuals with MCI had increased oxidative damage before the onset of symptomatic dementia by measuring 8,12-iso-iPF_2α_-VI levels in urine, plasma and cerebrospinal fluid using gas chromatography–mass spectrometry [16]. Arimon et al. demonstrated that local infusion of oxidizing agents into the hippocampus of wild-type mice increased local Aβ_42_ levels in the interstitial fluid, suggesting that oxidative stress is located upstream of Aβ pathology in AD [17]. Baldeiras et al. conducted a longitudinal study on 70 MCI patients and demonstrated that the accumulation of oxidative damage may start in presymptomatic phases of AD pathology and that progression to AD might be related to depletion of antioxidant defenses such as the oxidized/reduced GSH ratio and vitamin E [18].

### 2.2. Amyloid, Neurofibrillary Tangle and Oxidative Stress

Extracellular formation of senile plaques composed of Aβ is one of the hallmarks of AD pathology. Aβ is generated from APP though sequential cleavage by β and γ-secretases. Metal ions such as copper and zinc in the synaptic cleft of some neurons are supposed to play an important role in Aβ aggregation. Amyloid plaques also have a high content of these, and other, metal ions. Since these metal ions are involved in ROS production, aggregated Aβ takes part in ROS production [16]. Felica et al. showed that Aβ oligomers stimulated excessive formation of ROS through a mechanism requiring the activation of the N-methyl-D-aspartate (NMDA) receptor by using hippocampal neuronal cultures [19]. On the contrary, ROS themselves trigger Aβ generation by enhancing the amyloidogenic pathway [20]. Mitochondrial dysfunction and subsequent ROS production in a cell model induced by using rotenone and antimycin increased Aβ production [21]. In animal models, Aβ levels were enhanced by inhibiting complex I [21]. These findings suggest that there is bidirectional interplay between ROS and Aβ.

NFTs are another pathological hallmark of AD, and the formation of NFTs is thought to be linked closely to neuronal dysfunction in AD. NFTs are composed of a highly phosphorylated form of microtubule-associated protein tau [22]. Oxidative stress contributes to phosphorylation and the formation of NFTs [23]. By using an in vitro model of chronic oxidative stress through inhibition of glutathione synthesis with buthionine sulfoximine, Su et al. demonstrated that chronic oxidative stress increased levels of tau phosphorylated at the PHF-1 epitope in a time-dependent manner [24]. They also reported that a fragment of tau protein possessed copper reduction activity and initiated the copper-mediated generation of H_2_O_2_ [25].

### 2.3. Mitochondrial Dysfunction

The mitochondrion is an essential organelle that produces ATP through aerobic oxidative phosphorylation for sustaining cellular functions and survival, also serves as a regulator of cellular calcium concentration, and is a major generator of ROS. Therefore, dysfunctional mitochondria cause the loss of ATP, cellular calcium dysregulation, apoptosis and oxidative stress. O_2_^•−^, which is the proximal mitochondrial ROS, is produced by the one-electron reduction of molecular oxygen. Most of the O_2_^•−^ generated by intact mammalian mitochondria is produced by complex I, followed by complex III [26]. The production of O_2_^•−^ increases when the mitochondria are not making ATP and consequently have a high proton-motive force and a reduced coenzyme Q pool, and when the NADH/NAD^+^ ratio in the mitochondrial matrix is high [27].

Mitochondrial impairment is a common feature of the aging process [28] and AD [20]. Several mitochondrial functions decline with age, causing increased ROS production, mtDNA damage, changes in membranes and electrolytes and decreased recovery of damaged mitochondria [28]. Fluorodeoxyglucose positron emission tomography revealed reduced glucose metabolism in living AD patients, suggesting mitochondrial dysfunction [29]. In postmortem brains from AD patients, mitochondrial deoxyribonucleic acid (DNA) was damaged compared with the age-matched healthy control. Mitochondrial enzyme complexes are reduced in AD including cytochrome c oxidase, the pyruvate dehydrogenase complex, and the α-ketodehydrogenase complex, possibly explained by depletion of the mtDNA encoding subunits of such enzymes [30]. Manczak et al. reported that in APP transgenic AD model mice, expression of the mitochondrial fission genes *Drp1* and *Fis1* increased, expression of mitochondrial fusion genes *Mfn1*, *Mfn2*, *Opa1* and *Tomm40* decreased, and that Drp1 interacted with the Aβ monomer and oligomer, suggesting that increased production of Aβ and the interaction of Aβ with Drp1 are crucial factors in mitochondrial fragmentation, abnormal mitochondrial dynamics and synaptic damage [31].

### 2.4. Lipid Peroxidation

Lipid peroxidation occurs in the AD brain and is most prominent where degenerative changes are most pronounced [32]. A meta-analysis performed by Schrag et al. provided evidence of increased oxidative stress in serum, erythrocytes and circulating lymphocytes in AD, particularly in the lipid compartment [33]. Lipid peroxidation consists of a cascade of reactions, which causes the degradation of lipids mediated by free radicals. Free radicals abstract an allylic H from a methylene group in the acryl chain of phospholipids, followed by rearrangement of the double bonds to the conjugate diene form, producing a carbon-centered alkyl radical. When the alkyl radical reacts with paramagnetic molecular oxygen, a peroxyl radical is produced, which abstracts another allylic H atom to initiate a self-perpetuating chain reaction that ultimately leads to a variety of cyclic peroxides and hydroperoxides. Hydroperoxides can be further degraded to produce malondialdehyde (MDA), 4-hydroxynonenal (4-HNE) and acrolein, which can cause irreversible modification of phospholipids. Peroxidation of membrane lipids affects a variety of functions resulting in increased rigidity, decreased activity of membrane-bound enzymes, impairment of membrane receptors and altered permeability. 4-HNE binds to both nicastrin and beta-site amyloid precursor protein cleaving enzyme (BACE), differentially affecting γ- and β-secretase activity, suggesting that this naturally occurring product of lipid peroxidation may trigger the generation of toxic Aβ species [17]. Markers of lipid peroxidation are elevated in AD patients [34]. Membrane-associated oxidative stress occurs in association with the alterations in lipids, and exposure of the hippocampus to Aβ induces membrane oxidative stress and the accumulation of ceramide species and cholesterol [35].

### 2.5. Protein Oxidation

Protein oxidation also plays an important role in AD. Protein carbonyls generated by the oxidation of amino acids increased in AD brains [36]. Oxidative modification of proteins such as unfolding, conformational changes, protein–protein cross linking due to dityrosine formation, tyrosine halogenation and nitration and protein carbonylation can cause the loss of protein function, resulting in cell death [37]. Markers of protein oxidation such as carbonyls, dityrosine and 3-nitrotyrosine were elevated in the hippocampus and inferior parietal lobule of AD patients compared with age-matched controls [38].

Using a proteomics approach, Castegna et al. demonstrated that creatine kinase (CK) BB, ubiquitin carboxy-terminal hydrolase L-1, glutamine synthetase (GS), dihydropyrimidinase-related protein 2,α-enolase and heat shock cognate 71 were specifically oxidized in AD [39,40]. One consequence of oxidized CK is decreased availability of ATP in synaptic terminals, areas of the neuron that are probably most vulnerable and involved early in oxidative neurodegeneration in AD [40]. Impaired GS could reduce astrocyte protection against glutamate excitotoxicity to neurons [41].

### 2.6. Glycoxidation

Diabetes mellitus increases the risk of AD [42,43] through several mechanisms such as decreased Aβ clearance [44], cerebrovascular changes [45] and oxidative stress [46]. Advanced glycation endproducts (AGEs) are a group of heterogeneous compounds increasingly formed non-enzymatically by the Maillard reaction under hyperglycemic conditions [47]. Intracellular deposits of AGEs increased in both neurons and astrocytes of AD patients, and many neurons showed the co-localization of AGEs with hyperphosphorylated tau and nNOS [48].

The receptor for AGE (RAGE) is highly expressed in diabetes and the increased expression of RAGE was associated with increased oxidative and inflammatory stress [49]. RAGE expression levels in AD brains were higher than in control brains, and RAGE was present in neurons, glia and microglia in the hippocampus and cortex [50]. Wautier et al. demonstrated that enhanced oxidative stress by the AGE-RAGE signaling pathway is, at least in part, contributed by NADPH oxidase activation [51]. In addition, ligation of RAGE by AGEs resulted in the suppression of antioxidants such as GSH and ascorbic acid [52]. Askarova et al. demonstrated that Aβ binding to RAGE activated NADPH oxidase in endothelial cells and astrocytes, causing oxidative stress [53].

### 2.7. DNA Damage

ROS, especially ^•^OH, reacts with DNA by adding double bonds to the DNA base and by abstraction of an H atom from the methyl group of thymine and each C-H bond of 2′-deoxyribose [54], leading to strand breaks, as well as DNA–DNA and DNA–protein cross-linking [55]. 8-Hydroxy-2′-deoxyguanosine (8-OHdG) is a major form of DNA damage induced by ROS and is regarded as a marker of DNA oxidation. Nunomura et al. analyzed autopsy brains to demonstrate that the levels of neuronal 8-OHdG decreased exponentially as the Aβ burden in AD increased, noting a similar pattern of exponential decrease in neuronal 8-OHdG with increasing disease duration, suggesting that oxidative DNA damage is the earliest event and that AD is associated with compensatory changes that reduce damage from ROS [56].

Although the brain is the most affected in AD, Mecocci et al. demonstrated that the level of 8-OHdG was elevated in peripheral lymphocytes of AD patients [57]. Using comet assay analysis, Migliore et al. revealed a significantly higher level of primary DNA damage in leukocytes of AD and also of MCI patients compared to control individuals, suggesting that DNA damage is an earlier event in the pathogenesis of AD [58]. Moslemnezhad et al. also demonstrated that the plasma level of 8-OHdG was significantly higher in AD than in the control, while the amount of total antioxidants was significantly lower in patients compared to controls [59]. Isobe et al. revealed that the concentration of 8-OHdG in the CSF of AD patients was significantly higher than in the CSF of controls, and was positively correlated with the percentage of coenzyme Q10 and the duration of the illness [60].

### 2.8. Altered Antioxidant Defense

Progressive changes in oxidative stress defense mechanisms during the progression from MCI towards severe AD have been reported [61]. Antioxidants such as glutathione, GPx, GST and SOD significantly declined in the mitochondrial and synaptosomal fractions in the postmortem frontal cortex of MCI and AD patients [62]. A reduction of GSH was also demonstrated in the hippocampus and frontal cortex of living MCI and AD patients by using proton-magnetic resonance spectroscopy [63]. By using APP23 transgenic mice, Bayer et al. noted that chronic APP overexpression per se reduced SOD1 activity in the transgenic mouse brain, which could be restored to normal levels after Cu treatment [64].

Nrf2 is an antioxidant transcription factor. In unstressed conditions, Nrf2 in the cytoplasm is negatively regulated by Keap1. In oxidative stress, Nrf2 is stabilized and accumulates in the nucleus and activates its target genes such as GST and Heme oxygenase 1 (HO-1) [65]. Ramsey et al. reported that Nfr2 is predominantly cytoplasmic in hippocampal neurons in hippocampal neurons in AD, while it is expressed in both the nucleus and the cytoplasm in normal hippocampi with predominant expression in the nucleus, suggesting that Nrf2 does not respond properly to oxidative stress in AD neurons [66]. Using AD model *App^NL-G-F/NL-G-F^* knock-in mice and a natural compound, 6-(methylsulfinyl)hexyl isothiocyanate, which mildly activated Nrf2 signaling, the induction of Nrf2 ameliorated cognitive impairment in the AD model mouse by suppressing oxidative stress and neuroinflammation [67].

### 2.9. Amyloid Clearance and Oxidative Stress

Ultrastructural studies demonstrated characteristic and extensive angioarchitectural distortion of cerebral capillaries in AD [68,69]. Low-density lipoprotein receptor-related protein 1 (LRP-1) is the primary moiety responsible for the efflux of Aβ from the brain to the blood across the blood–brain barrier (BBB). In an autopsy of AD brains, the levels of 4-HNE bound to transmembrane LRP-1 had significantly increased in the hippocampus, while the levels of LRP-1-3-nitrotyrosine had not, suggesting that Aβ impaired its own efflux from the brain by oxidation of its transporter LRP-1, leading to increased Aβ deposition [70]. On the contrary, RAGE not only causes oxidative stress described above, but also promotes influx of circulating Aβ across the BBB [71]. FPS-ZM1, a specific RAGE inhibitor, downregulated Aβ influx across the BBB, decreased hippocampal Aβ, inhibited NF-κB signaling and reduced apoptosis in db/db mice [72].

### 2.10. Inflammation

The interaction between oxidative stress and inflammation contributes to AD pathology [73]. Damaged neurons, insoluble Aβ peptide deposits and NFTs stimulate inflammation in the AD brain [74]. Microglia are resident macrophages in the central nervous system [75] and are principle immune effectors [76]. Inflammatory responses are mediated by the activation of microglia [77]. In AD brains, microglia are activated and are attracted to and surround senile plaque [78]. Quantitative in-vivo measurements of glial activation with PET and carbon-11-labelled (*R*)-PK11195 demonstrated that AD patients showed a significant increase of microglial activation in entorhinal, temporal and cingulate cortexes [79]. Fibrillar Aβ-stimulated microglia release ROS [74]. The primary source of ROS and the source of widespread oxidative damage found in AD brains is microglial NADPH oxidase [76]. Astrocytes are also activated in the AD brain [80]. Similar to microglia, astrocytes release cytokines, interleukins, NO^•^ and other potentially cytotoxic molecules upon exposure to Aβ [81]. Aβ upregulated both pro- and anti-inflammatory cytokines including IL-1β, IL-6, transforming growth factor-β and IL-10 [82]. In turn, neuro-inflammation-induced oxidative stress increases the expression of Aβ [73].

### 2.11. Chronic Cerebral Hypoperfusion

Chronic cerebral hypoperfusion (CCH) is ubiquitous in elderly AD patients, and can play pivotal roles in triggering and exacerbating the pathophysiological progress of AD. Our previous studies revealed that white matter hyperintensity (WMH) was observed in more than 88% of AD patients by magnetic resonance imaging (MRI) [83], and that high grade WMH was a risk factor for MCI conversion to AD as well as low educational attainment, a low baseline mini-mental state examination (MMSE) score and parahippocampal gyrus atrophy [83]. Analysis of the Alzheimer’s Disease Neuroimaging Initiative public database revealed that Pittsburg compound B (PIB) positivity increased total WMH volume independently of the predicted AD diagnosis, that those diagnosed as having AD had greater WMH volume among PIB-positive subjects than normal control subjects and that both WMH and PIB status at the baseline conferred risk for future diagnosis of AD, suggesting that WMH contributes to the presentation of AD and may provide a second hit necessary for the clinical manifestation of the disease [84]. We revealed that neural oxidative stress and neuroinflammation were enhanced in AD model mice with CCH and that edaravone, a free radical scavenger, significantly improved motor and cognitive deficits, attenuated neuronal loss, reduced Aβ/phosphorylated tau (pTau) accumulation and alleviated neural oxidative stress and neuroinflammation in the AD mouse model with CCH [85]. Additionally, CCH greatly enhanced the number of Aβ oligomer-positive/pTau cells, the expression of peroxidation products (4-HNE and 8-OHdG), mitochondrial fission proteins (Drp1 and Fis1), and decreased the expression of mitochondrial fusion proteins (Opa1 and Mfn1) in the CTX and thalamus (TH) of AD model mice at 12 month of age, demonstrating that CCH shifted the balance in mitochondrial morphology from fusion to fission [86]. Furthermore, we investigated expressive changes of two main Aβ transport receptors, LRP-1 and RAGE, and revealed that CCH increased LRP-1 and RAGE expression in brain parenchyma, while a decrease of LRP1 and increase of RAGE were observed in vascular endothelial cells, suggesting double imbalances of Aβ efflux and influx transport-related proteins in the cortical blood vessel of AD mice. These neuropathological abnormalities were greatly ameliorated by edaravone [87].

## 3. Therapeutic Approach for Alzheimer’s Disease

There have not been any effective treatments to prevent, halt or reverse AD [88]. Currently available therapies with cholinesterase inhibitors such as donepezil hydrochloride, galantamine and rivastigmine or NMDA receptor antagonist memantine offer little more than short-term palliative effects [89]. Although several trials of amyloid-targeting therapy have been performed recently, they have yet to show satisfactory results.

Based on findings that oxidative stress plays a major role in AD, oxidative stress has been considered as a therapeutic target of AD [89]. The free radical scavenger edaravone, which was approved as a treatment of acute ischemic stroke [90] and amyotrophic lateral sclerosis [91], inhibited Aβ aggregation and Aβ-induced oxidation in vitro, and improved AD pathology and cognitive behavioral deficits of AD model mice [92]. Treatment with coenzyme Q10, a component of the mitochondrial electron transport chain, decreased brain levels of carbonyls, plaque area and number in the hippocampus and in the overlying cortex immunostained with an Aβ_42_-specific antibody, brain Aβ_42_ levels and levels of Aβ protein precursor β-carboxyterminal fragments, and improved performance in the Tg19959 mouse model of AD [93]. Melatonin has inhibitory effects on the formation of secondary β-sheet structures and amyloid fibril formation in vitro [94,95], and administering melatonin into Tg2576 AD model mice partially inhibited the time-dependent elevation of Aβ, reduced abnormal nitration of proteins, and increased survival [96]. Overexpression of SOD-2 reduced hippocampal O_2_^•−^ and prevented memory deficits in the Tg2576 mouse model of AD [97].

In spite of such favorable preclinical study outcomes, previous antioxidative components including a single antioxidative supplement such as vitamin C, vitamin E or their mixtures [98,99,100] did not clearly show a therapeutic effect on cognitive decline in AD [101,102,103,104], even though some of the clinical studies suggest a possible therapeutic effect of these compounds. However, we recently reported that novel supplements such as Twendee X^®^ and tocotrienols can be beneficial for AD patients.

## 4. Twendee X

Twendee X^®^ (TwX; TIMA Japan, Osaka, Japan) is a patented supplement containing coenzyme Q10, niacin amide, L-cystine, ascorbic acid, succinic acid, fumaric acid, L-glutamine and riboflavin, having stronger antioxidant and anti-inflammatory effects than single antioxidant vitamins [105]. Inufusa et al. characterized the antioxidant properties of TwX, assessing parameters of the redox state following the induction of oxidative stress by H_2_O_2_ in HepG2 cells. In HepG2 cells, H_2_O_2_ exposure increased ROS at the mitochondrial (69%) and cellular level (68%), reduced natural antioxidant enzymatic activity with reduced Mn (32%) and Cu/ZnSOD1 (31%) activities and increased H_2_O_2_ scavengers with a 31% increase in total glutathione activity. TwX modulated H_2_O_2_ oxidative activity by reducing the level of ROS produced in the two compartments, increasing direct antioxidant defense at the mitochondrial and cellular levels, and reducing H_2_O_2_-induced scavenging activity by reducing GSH activity [106]. The therapeutic effect of TwX was also examined in nude mice that underwent inoculation of colon or gastric cancer cells: TwX reduced tumor growth, increased antioxidants measured by the d-ROMs test, and reduced natural killer cell activity, suggesting that TwX significantly reduced tumor growth and inhibited metastasis by reducing oxidative stress [107]. In ischemic stroke model mice, we demonstrated that pretreatment of TwX (20 mg/kg/d for 14 days) reduced infarct size as well as the expression of both oxidative stress markers such as 8-OHdG, 4-HNE and Nε-(carboxymethyl) lysine (an advanced glycation end product) and inflammatory markers such as Iba-1, tumor necrosis factor α (TNF-α) and monocyte chemotactic protein [108,109].

We also investigated the therapeutic effect of TwX on cognitive function, Aβ pathology, oxidative stress and inflammation in a novel AD mouse model with CCH. TwX treatment (20 mg/kg/d, from 4.5 to 12 months) significantly ameliorated cognitive deficit, amyloid-β, tau and α-synuclein pathology, neuronal loss and neurovascular dysfunction, and this was accompanied by the attenuation of both oxidative stress (4-HNE, 8-OHdG) and inflammatory markers (NACHT, LRP and PYD domains-containing protein 3 (NLRP3), caspase-1, IL-1β, Iba-1 and TNF-α) [110].

In a multicenter, randomized, double-blind and placebo-controlled prospective interventional study, TwX showed a significantly higher score of the mini-mental state examination at 6 months compared with the placebo, and also a significant improvement of the Hasegawa dementia scale-revised score from baseline at 6 months, suggesting that a strong antioxidative therapy might be a useful way to prevent the conversion of MCI to AD [111].

## 5. Tocotrienols

**Figure 1:**
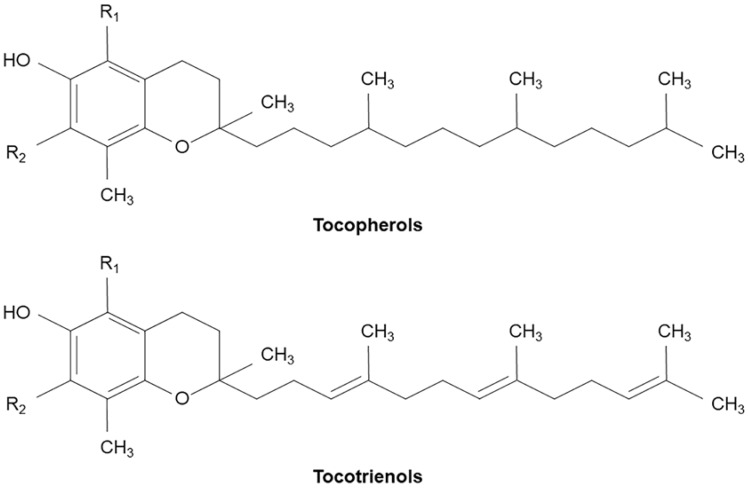
Molecular structure of tocopherols and tocotrienols.

Vitamin E is a lipid component of biological membranes and a potent antioxidant consisting of two categories: tocopherols and tocotrienols, each with 4 α, β, γ and δ analogs. Both tocopherols and tocotrienols consist of a chromanol ring and a 15-carbon tail, but tocotrienols differ from tocopherols by the presence of three unsaturated bonds in the hydrocarbon tail (Figure 1) [112]. Tocopherols are found in lipid-rich regions of cells such as mitochondrial membranes, fat depots and lipoproteins such as low-density lipoprotein cholesterol [113]. Vitamin E detected in the brain is virtually only α-tocopherol [114]. Orally supplemented α-tocotrienols were effectively delivered to most tissues, including the brain, in mice with α-tocopherol deficiency [115]. Epidemiological studies revealed that AD and MCI had lower levels of total tocopherols, total tocotrienols and total vitamin E compared with cognitively normal subjects [116,117].

Oral vitamin E supplement mainly consists of α-tocopherol, which was unable to prevent cognitive decline [98,99,100]. However, the antioxidant activity of α-tocotrienols is higher than that of α-tocopherols [114]. The antioxidative activity of tocopherols is related to the scavenging of free radicals in unsaturated lipid [118]. Compared to tocopherols, tocotrienols are widely distributed in the phospholipid bilayer and easily interact with lipids due to the unsaturated bonds of the hydrocarbon tail (Figure 1) [112]. An increasing number of studies have shown that tocotrienols possess additional beneficial pharmacological actions such as inhibited platelet aggregation [119], monocytic adhesion and cholesterol-lowering activity, which are independent of their antioxidant properties. Khanna et al. demonstrated that tocotrienols blocked glutamate-induced death by suppressing early activation of c-Src kinase and 12-Lox [120]. Gopalan et al. demonstrated that mixed tocotrienols attenuated the progression of white matter lesions, indicating cerebral small vessel disease [121]. Ibrahim et al. demonstrated that treatment with a tocotrienol-rich fraction (TRF) dose-dependently inhibited the formation of Aβ formation fibrils and Aβ oligomers in vitro, and that daily TRF supplementation to AβPPswe/PS1dE9 double transgenic mice for 10 months attenuated Aβ immunoreactive depositions and thioflavin-S-positive fibrillar type plaques in the brain and eventually improved cognitive function [122,123].

Tocovid Suprabio^TM^ (Hovid, Perak, Malaysia) is a patented supplement that consists of 61.52 mg α-tocotrienol, 112.80 mg γ-tocotrienol, 25.68 mg δ-tocotrienol and 91.60 IU α-tocopherol in a capsule with a self-emulsifying system (Suprabio™), providing higher and more consistent absorption of tocotrienols. We reported that the neuroprotective effects of Tocovid Suprabio^TM^ in the ischemic stroke model mice were accompanied by amelioration of motor dysfunction and infarct volumes [124,125]. Tocovid Suprabio^TM^ significantly decreased the expression of oxidative stress markers (4-HNE, nitrotyrosine and 8-OHdG), advanced glycation markers (RAGE, carboxymethyl arginine (CMA) and carboxymethyl lysine (CML)) and apoptotic and autophagy markers (cleaved caspase-3 and LC3-II), and enhanced the expression of Nrf2 and multidrug resistance protein 1 (MRP1) accompanied by a decrease of the glutathione disulfide (GSSG)/GSH ratio [124]. In addition, Tocovid Suprabio^TM^ decreased the expression of inflammatory markers such as TNF-α, monocyte chemotactic marker-1 (MCP-1) and Iba-1, and improved the damage of neurovascular units including matrix metalloproteinase 9 (MMP9), IgG and collagen IV [125]. These studies obviously demonstrated that Tocovid Suprabio^TM^ treatment showed neuroprotective effects through antioxidative stress, antiapoptotic/autophagic and anti-inflammatory effects in the ischemic mouse brain.

A clinical trial reported that mixed tocotrienols attenuated the progression of white matter lesions in the human brain after 2 years [121] and that this was related with cognitive decline [126], suggesting that tocotrienols might prevent cognitive decline in AD.

## 6. Mitochondria-Targeted Antioxidants and Polyphenols

Plastoquinonyl-decyltriphenylphosphonium (SkQ1), mitoquinone mesylate (MitoQ) and astaxanthin are mitochondria-targeted antioxidants [127,128]. SkQ1 increased behavioral activity, and reduced destructive changes in mitochondria, pathological accumulation of AβPP, Aβ, hyperphosphorylation of tau-protein and hippocampal Aβ_40_ and Aβ_42_ protein levels in AD model rats [129,130]. MitoQ attenuated Aβ-neurotoxicity in the cortical neuron and prevented increased production of ROS, loss of mitochondrial membrane potential, cognitive decline, Aβ accumulation, astrogliosis, synaptic loss and caspase activation in AD model mice [131]. In a randomized, placebo-controlled, double-blind, crossover design study, MitoQ improved vascular function in healthy older adults [132]. Astaxanthin reduced cognitive impairment, soluble Aβ_42_, insulin receptor substrate-1 S307 phosphorylation, glycogen synthase kinase-3β phosphorylation, in AD model rats [133]. A composite supplement containing astaxanthin and sesamin improved permeability psychomotor speed and processing speed of MCI subjects in a randomized, double-blind, placebo-control trial [134].

Polyphenols are also expected to be beneficial for AD patients [135]. Curcumin is a polyphenol from *Curcuma longa*, and has an antioxidant property as well as anti-inflammatory and antiamyloid effects [136]. Curcumin treatment improved the behavioral symptoms in AD patients [137]. Maiti et al. reported that solid lipid curcumin particles (SLCP) provide more antiamyloid, anti-inflammatory and neuroprotective outcomes than natural curcumin, and intraperitoneal injection of SLCP decreased Aβ plaque loads, pyknotic or tangle-like neurons, and reduced glial fibrillary acidic protein and Iba-1 immunoreactivity more strongly than natural curcumin [138]. Resveratrol is a polyphenol found in red grapes, red wine and other plant foods. Resveratrol treatment significantly prevented memory loss, reduced the amyloid burden and increased mitochondrial complex IV protein levels in the mouse brain mainly through Sirtuin 1 and adenosine monophosphate-activated protein kinase pathways in AD model mice [139]. In a randomized, double-blind, placebo-controlled trial, oral resveratrol supplementation stabilized CSF Aβ_40_ and plasma Aβ_40_ levels, which declined significantly in the placebo group [140].

## 7. Conclusions

**Figure 2:**
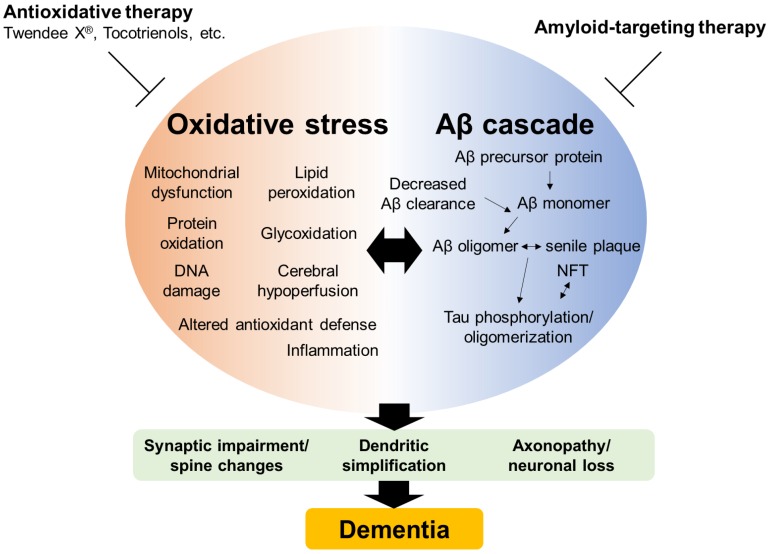
Schematic illustration of oxidative stress and amyloid β (Aβ) cascade in Alzheimer’s disease. There is interplay between oxidative stress and the Aβ cascade, resulting in neuronal dysfunction and death. Antioxidative supplements such as Twendee X^®^ and tocotrienols might be hopeful for preventing dementia.

There is considerable demand for effective interventions to prevent cognitive decline in AD, and oxidative stress can be a therapeutic target of AD. Novel antioxidative supplements might be hopeful antioxidative supplements for preventing dementia (Figure 2).

## Abbreviations

AD	Alzheimer’s disease
AGE	advanced glycation end-product
ALS	amyotrophic lateral sclerosis
ApoE	apolipoprotein E
APP	amyloid precursor protein
ATP	adenosine triphosphate
Aβ	amyloid β
BACE	beta-site amyloid precursor protein cleaving enzyme
BBB	blood-brain barrier
CCH	chronic cerebral hypoperfusion
CK	creatine kinase
CMA	carboxymethyl arginine
CML	carboxymethyl lysine
CSF	cerebrospinal fluid
CTX	Cortex
GPx	glutathione peroxidase
GS	glutamine synthetase
GSH	Glutathione
GSSG	glutathione disulfide
GST	glutathione-*S*-transferase
HNE	Hydroxynonenal
H_2_O_2_	hydrogen peroxide
LRP-1	low-density lipoprotein receptor-related protein 1
MCI	mild cognitive impairment
MCP-1	monocyte chemotactic protein-1
MDA	Malondialdehyde
MitoQ	mitoquinone mesylate
MMP9	matrix metalloproteinase 9
MMSE	mini-mental state examination
MRI	magnetic resonance imaging
MRP1	multidrug resistance protein 1
NADPH	nicotinamide adenine dinucleotide phosphate
NFT	neurofibrillary tangles
NLRP3	NACHT, LRP and PYD domains-containing protein 3
NMDA	N-methyl-D-aspartate
O_2_^•-^	superoxide anion
^•^OH	hydroxyl radical
PET	positron emission tomography
PIB	Pittsburg compound B
pTau	phosphorylated tau
RAGE	receptor for advanced glycation end-product
RNS	reactive nitrogen species
ROS	reactive oxygen species
SkQ1	plastoquinonyl-decyltriphenylphosphonium
SLCP	solid lipid curcumin particles
SOD	superoxide dismutase
TH	Thalamus
TNF-α	tumor necrosis factor-α
TRF	tocotrienol-rich fraction
TwX	Twendee X
WMH	white matter hyperintensity
8-OHdG	8-hydroxy-2′-deoxyguanosine
